# Validation of an instrument to determine oral health knowledge, attitudes, and practices during pregnancy

**DOI:** 10.1186/s12903-021-01898-1

**Published:** 2021-10-30

**Authors:** María de los Ángeles Ramírez-Trujillo, María del Carmen Villanueva-Vilchis, Fátima del Carmen Aguilar-Díaz, Javier de la Fuente-Hernández, Daniel Demétrio Faustino-Silva, Luis Alberto Gaitán-Cepeda

**Affiliations:** 1 Department of Public Health, National School of Higher Studies, Leon Unit, National Autonomus University of Mexico (UNAM), Blvd. UNAM #2011, Predio El Saucillo y El Potrero, Comunidad de los Tepetates, 37684 León, Guanajuato México; 2 Grupo Hospitalar Conceição (GHC), Porto Alegre, Brazil; 3 Laboratory of Oral Pathology, Posgratuate and Research Division, Dental School, National Autonomus University of Mexico, México City, México

**Keywords:** Validation study, Oral hygiene, Dental health surveys, Prenatal education, Pregnant women

## Abstract

**Background:**

Oral health of the mother-infant dyad is important to preserve general health. However, there are few instruments in Spanish for the evaluation of knowledge, attitudes and practices that determine this construct. Therefore, this research aimed to develop and evaluate the psychometric properties of the Maternal Oral Health Knowledge, Attitudes and Practices Questionnaire (CAPSOM in Spanish).

**Methods:**

In this instrument development study that carried out in 2018–2019, involving pregnant women between the ages of 18 and 45 in the city of Leon, Guanajuato, Mexico. The sample size was calculated based on 10 women per questionnaire item (n = 10 k). The study used Cronbach’s alpha, the modified Lawshe test of validity criteria, factor analysis, and the level of difficulty and discrimination of the items.

**Results:**

207 women took part with their signed, informed consent (25 ± 6 years). The internal consistency of the instrument, both total and by dimension was α = 0.70, α = 0.66 knowledge, α = 0.74 attitudes, and α = 0.66 practices. Values of Content Validity Ratio’ ≥ 0.60 were obtained for the final 10 items and Content Validity Index’ = 0.90. The average difficulty index of items was 0.40, and there were significant differences (Kruskall–Wallis, *p* < 0.001) in the discrimination test. Factor analysis demonstrated three main components.

**Conclusions:**

A valid and reliable 10-item Spanish questionnaire was designed to measure pregnant women’s oral health knowledge, attitudes, and practices.

## Background

The wellbeing of the mother-infant dyad has a position of vital importance in public health, since it is a fundamental indicator of health and social inequalities [1]. A poor oral condition has direct implications on general health. For example, it affects children's growth, ability to concentrate, hours of sleep, and even their socialization, in such a way that we must address all the factors related to that condition, including those related to the role and health of the mother [2]. On the other hand, there is a strong relationship between homeostasis, oral biofilm, and general health in such a way that during pregnancy periodontal disease might be considered as a risk factor for preterm birth, low birthweight, and preeclampsia [3]. During pregnancy, the mother and her child, face diverse health risks, so that the identification of the factors involved in the development of oral diseases, and the attendant preventive and prophylactic measures, are the first steps in oral health [4].

It is an established fact that mothers play a fundamental role as facilitators in achieving good oral health by their children. Thus, improvement in their knowledge and attitudes towards oral habits, will undoubtedly help improving the oral health of their children [4–7]. Therefore, the transmission of conducts and behaviors from mother to child has repercussions for the latter’s oral health, specifically during the period of its first thousand days of life. For this reason, understanding the attitudes and knowledge on the subject of oral care, as well as determining the oral health practices of pregnant women, is important, as it will reveal their state of oral health during the course and conclusion of their pregnancies [3, 8, 9].

Despite the importance of learning about and determining pregnant women’s behaviors, and the fact that the last decade has seen the publication of information relating to oral self-care for expecting mothers, the instruments used for collecting this critical data are scarce. Some of the reports on the instruments available for the evaluation of oral health KAPs in pregnant women do not describe their psychometric characteristics, as many come from pilot tests with limited sample sizes [10–12]. On the other hand, several of the questionnaires designed for this purpose do not comprehensively assess knowledge, attitudes, and practices [13–18]. In addition, it is necessary to include some aspects related to cultural beliefs about oral health related to pregnancy that can be decisive for the wellbeing of the mother–child binomial and that are not addressed in most of the published instruments [19]. Given this, and with the aim of developing a tool for data collection that is valid, reliable and easily administered.

## Methods

The aim of this research was to develop and evaluate the psychometric properties of the Maternal Oral Health Knowledge, Attitudes and Practices of pregnant women in the area of oral health: the CAPSOM (*Conocimientos, actitudes y prácticas de salud oral materna*).

The study was carried out in two phases: (1) Design and construction of the instrument to establish the knowledge, attitudes, and practices of pregnant women about oral health; and (2) validation of the instrument. The research protocol was approved by the Research Ethics Commission of the National School of Higher Studies (ENES), Leon Unit, of the National Autonomous University of Mexico (UNAM) (CEI.18_013_S1).

### Phase 1: Design and construction of the CAPSOM instrument

In preparation for the design of the instrument, two public health experts reviewed the scientific literature on and conceptual models of mother–child oral health [19–21], along with the recommendations contained in the pediatric guidelines issued by the American Academy of Pediatric Dentistry (AAPD) [22]. From the information collected, 20 questionnaire items were formulated in relation to oral health knowledge, attitudes, and practices regarding visits to the dentist, oral diseases during pregnancy, hygiene, calcium, and tooth loss.

### Face validity

The 20 items were subjected to expert’s face validity analysis, which resulted in the removal of seven items that were considered either repetitive or poorly constructed. This evaluation was made from the consensus of the two experts, submitting it to the evaluation of a third expert in pediatric dentistry to resolve the disagreements. The remaining 13 items were subjected to evaluation of face validity by the users on their order, grammar, clarity, and relevance through a pre-test involving five pregnant women attending a teaching clinic on pediatric dentistry (ENES-UNAM, León, Guanajuato, México). Based on the observations collected from the participants, linguistic-cultural adjustments were made to any items considered hard to understand.

Thus, the initial instrument consisted of 13 items split between 3 dimensions: (A) practices; (B) knowledge; and (C) attitudes.

### Phase 2: Validation and reliability of the instrument

The cross-sectional study was carried out between October 2018 and April 2019. The study included pregnant women between 18 and 45 years of age attending prenatal checkups at three gynecological and obstetric care centers in the state social security system in the city of Leon, Guanajuato, Mexico. To be included in the study, the women had to be capable of answering the questionnaire by themselves. Those participants who did not complete the questionnaire and who voluntarily withdrew from the study were eliminated.

The sample size was calculated based on a minimum of ten women per questionnaire item in order to perform a factor analysis of an instrument consisting of 10 items (*n* = 10 k) [23]. The following demographic data were collected: age; educational level (from none to primary, secondary, high school or higher), occupation (homemaker, employee, professional, student, other), marital status (single, cohabiting, married, divorcee, widow).

### Data collection

Once the modifications based on the pre-test were completed, and subsequent to a request for and receipt of informed consent, the participants were interviewed in the Education Room at the facilities of each of the centers included. Before filling out the questionnaire, they were given instructions on the procedure, stressing the privacy and confidentiality of the data and requesting that they provide sincere responses.

### Reliability (internal consistency)

The Cronbach’s alpha reliability coefficient was used to establish the internal consistency values for the instrument as a whole, by dimension and for each separate item.

### Content validity by experts

Content validity by experts was established by using the modified Lawshe test [24]. Ten public oral health experts from five Latin American higher education institutions took part (the National Autonomous University of Mexico’s National School of Higher Studies, Leon Unit, and its Dental School, the CES University of Medellín, Colombia, Chile’s Andrés Bello University and the University of Sao Paulo, Brazil). Each item was classified as either “essential”, “useful but not essential”, or “not necessary”. In addition, the Content Validity Ratio (CVR’) was obtained for each item, this being defined as the proportion of essential agreements in relation to the total number of items. The acceptable value was set at ≥ 0.60, in accordance with the recommendations of Tristán [24]. Finally, the Content Validity Index (CVI’) of the entire instrument was calculated.

### Item difficulty index (D) and Item discrimination level (d)

The quality of the items was established via a numerical expression of difficulty (*D*) in which 0 indicated high difficulty and 1 low difficulty, with acceptable values falling between 0.20 and 0.80. The item discrimination level (*d*) evaluated the degree to which the question helped to increase the estimated differences between those which achieved a relatively high score on the test and those with a relatively low one. For this purpose, in order to compare the highest and lowest values, the tertile values for each dimension of the scale were obtained using the Kruskall Wallis test. The existence of a statistically significant difference was interpreted as good discrimination by the items [25, 26].

### Factor analysis

In order to continue with the evaluation of construct validity, factor analysis was then performed. This verified the suitability of the sample respecting the correlation between the variables included by means of the Kaiser–Meyer–Olkin test and Bartlett’s Sphericity test [27].

To extract the factors, the principal components method was employed and, in order verify the relation between these, the Varimax orthogonal rotation method was used. It was estimated that there was an adjusted factorial charges with the values ≥ 0.40. Lastly a calculation was made of the variance explained by the solution [27].

## Results

A total of 207 pregnant women with an average age of 25 ± 6 (in the 18–45 range) took part in the study. In terms of educational level, the most common was secondary, followed by high school. The occupation most frequently reported was homemaker, while the most common marital status was cohabiting (Table 1).

**Table 1:** Description of the population by sociodemographic characteristics. Leon, Guanajuato, Mexico, 2019. (n = 207)

Sociodemographic characteristic	n	(%)
*Education*		
None	7	3.40
Primary	35	16.90
Secondary	80	38.60
High school	49	23.70
Higher	36	17.40
Total	207	100
*Occupation*		
Homemaker	132	63.80
Employee	40	19.30
Professional	16	7.70
Student	8	3.90
Other	11	5.30
Total	207	100
*Marital status*		
Single	42	20.20
Cohabiting	84	40.60
Married	78	37.70
Divorcee/widow	3	1.50
Total	207	100

## Content validity by experts

With regard to the CVR’ (Table 2), values above 0.60 were obtained for 10 items. However, the statements, “Teeth should be brushed once or twice a day during pregnancy”, “Going to the dentist during pregnancy poses a risk for my baby and for me”, and “It is very important to monitor your oral health during pregnancy” received unacceptable values. Once these items were removed, the result was the final 10-item version of the CAPSOM, whose CVI’ was 0.90.

**Table 2:** Content validity, difficulty, and discrimination per item on the CAPSOM. Leon, Guanajuato, Mexico, 2019. (n = 207)

Item	Dimension	Attribute	CVR’^a^	A/R^b^	D^c^	d^d^
1	Knowledge	Issues with tooth decay and bleeding gums can get worse during pregnancy	1	A	0.45	*p* < 0.001
2		Gum problems can affect my pregnancy and create problems with my baby’s birth	0.9	A	0.72	
3		It is inevitable to lose a tooth during pregnancy	0.8	A	0.5	
4		My baby’s development will extract calcium from my teeth	0.8	A	0.33	
–		Teeth should be brushed one or more times a day during pregnancy	0.5	R	**–**	**–**
–		Going to the dentist during pregnancy poses a risk for my baby and me	0.3	R	–**–**	**–**
5	Attitudes	Hygiene measures are important to minimize any oral complications that may arise during the pregnancy	0.8	A	0.35	*p* < 0.001
6		It is important to go to the dentist before, during and after the pregnancy	0.9	A	0.36	
–		It is very important to monitor your oral health during pregnancy	0.4	**R**	**–**	**–**
7	Practices	I brush my teeth twice or more times a day	1	A	0.41	*p* < 0.001
8		I use other methods of oral hygiene such as mouthwash, flossing, etc	1	A	0.35	
9		I have received information from a dental professional about oral health care during my pregnancy	0.8	A	0.21	
10		I have visited a dentist during my pregnancy	1	A	0.32	
			CVI’^e^ 0.90		Total D^f^ 0.40	

^a^CVR’: content validity ratio for 10 experts (acceptable value > 0.60)

^b^A/R: item accepted/item rejected according to the value obtained from the CVR’

^c^D: difficulty index for the item (acceptable value 0.20 to 0.80)

^d^d: discrimination level per item (Kruskal–Wallis, 0.05)

^e^CVI’: Content Validity Index (acceptable value > 0.58)

^f^Total D: average difficulty index of the item (acceptable values close to optimum difficulty 0.50)

### Content validity

The average difficulty index of the items was 0.40. For the evaluation of the level of discrimination, a statistically significant difference (Kruskall Wallis, *p* < 0.001) was observed in all the variables included, indicating that the items discriminated well (Table 2).

Analysis produced values appropriate for KMO of 0.66 and for Bartlett’s Sphericity of *p* < 0.001, demonstrating that they confirmed the assumptions regarding the administration of the test. The factor analysis revealed the existence of three dimensions: knowledge, attitudes, and practices, whose total explained variance was 57.11%, this being the proportion of variance which the item scores can explain by means of the three factors identified.

Table 3 shows the loading factor on each dimension. The results show that factor one, which explains 25.10% of the variance, consists of items 7, 8, 9, and 10 in the practices dimension. Factor two explains 19.83% of the variance and contains the knowledge items 1, 2, 3, and 4. Lastly, factor 3 is composed of items 5 and 6, which correspond to the attitudes dimension, and explains 12.16% of the variance.

**Table 3:** Loading factor value of each questionnaire item in three components. Leon, Guanajuato, Mexico, 2019. (n = 207)

Item	Component		
	1	2	3
	Practices	Knowledge	Attitudes
Issues with tooth decay and bleeding gums can get worse during pregnancy	–	0.63	**–**
Gum problems can affect my pregnancy and create problems with my baby’s birth	–	0.76	**–**
It is inevitable to lose a tooth during pregnancy	–	0.76	**–**
My baby’s development will extract calcium from my teeth	–	0.65	**–**
Hygiene measures are important to minimize any oral complications that may arise during the pregnancy	**–**	–	0.85
It is important to go to the dentist before, during and after the pregnancy	**–**	–	0.85
I brush my teeth twice or more times a day	0.47	**–**	–
I use other methods of oral hygiene such as mouthwash, flossing, etc	0.73	**–**	–
I have received information from a dental professional about oral health care during my pregnancy	0.75	**–**	–
I have visited a dentist during my pregnancy	0.75	**–**	–
Loading factor > 0.4			
Varimax rotation			

### Reliability (internal consistency)

An analysis of internal consistency was performed which resulted in a total of 0.70 for ten items. Likewise, the Cronbach’s Alpha value was analyzed for each dimension of the questionnaire, with resulting values of α = 0.66 for knowledge, α = 0.74 for attitudes, and α = 0.66 for practices (Table 4), denoting moderate to acceptable internal consistency values for each of the items. Additionally, this analysis guaranteed the continued inclusion of each of the items since it was possible to verify that the removal of none of them increased the alpha value concerning the total value of the instrument.

**Table 4:** Internal consistency per item on the CAPSOM. Leon, Guanajuato, Mexico, 2019. (n = 207)

Dimension	Cronbach's alpha if an item is removed	Cronbach's alpha by dimension
Knowledge	0.63	0.66
	0.54	
	0.53	
	0.62	
Attitudes	0.59	0.74
	0.59	
Practices	0.65	0.66
	0.62	
	0.54	
	0.54	
Total		0.70

As a result of these analyses, the final version of the instrument consisted of ten items, with true/false responses for the four in the “knowledge” dimension, and a Likert scale (Completely disagree, Disagree, Neutral, Agree and Completely agree) for the six remaining items corresponding to the “attitudes” and “practices” dimensions. Appendix 1. Thus, the minimum score on the instrument is 0 and the maximum 28, and the higher the score, the greater the levels of knowledge, attitudes, and practices.

## Discussion

This project designed and validated an instrument to determine the pregnant women’s knowledge, attitudes, and practices regarding oral health during their pregnancies with the aim of creating a reliable and useful tool for the collection of data that would provide valuable information for the study of the oral health of the mother–child dyad, and ultimately establish strategies and plans for improving perinatal oral health, for both mothers and their newborn babies. The study of knowledge on dentistry for newborns has acquired enormous importance in recent decades. The development of this instrument took into account the various approaches to education on babies’ dental health, resulting in the inclusion of items related to mothers’ knowledge and perception of oral diseases during pregnancy, oral hygiene measures, tooth loss, calcium loss and the possible risks involved in attending dental appointments during pregnancy [28–31].

Concerning reliability, the minimum values obtained were 0.66 for Cronbach’s alpha in the dimensions of knowledge and practices. It has been suggested that, in newly developed instruments, values higher than 0.60 should be considered acceptable [32, 33]. For this reason, the values that we obtained for the entire questionnaire, as well as for each of the dimensions, are indicative of strong internal consistency.

With regard to content validity, it was decided that the evaluations carried out by the panel of experts should be done individually, and evidently in different physical spaces, thus avoiding any influence by the opinions of others, as happens with the Nominal Group Technique [34]. The inclusion of experts from various fields of expertise—e.g., university academics, clinicians, public health experts, pediatric dentists, experts in mother-infant interventions, and an expert in dentistry for babies—strengthened the constructs.

The Lawshe test, as modified by Tristán in 2008, was utilized as it makes it possible to make both an estimate of validity per item (CVR’) and an estimate for the instrument as a whole (CVI’) [24], as opposed to the method proposed by Delphi [35], which requires the participation of a larger number of experts and is more time-consuming, thereby resulting in a high number of withdrawals. Additionally, the modified Lawshe test makes it possible to obtain cutoff points according to the number of panelists, thereby facilitating the interpretation of the values. The wide range of differences between the experts can lead to difficulties in reaching a consensus [33, 36]. Therefore, given their acceptable CVR’ values, we believe that the items on the proposed instrument are sufficiently robust to measure the construct.

Regarding content validity, for this project, it was decided to include—prior to the factor analysis—an evaluation of the quality of the items by means of the index of difficulty and level of discrimination commonly used in education [33, 37]. In the instrument proposed here, the average difficulty index per item was found to be 0.40, a value close to optimum difficulty (0.50). Currently, these indices are introduced into the health context when dealing with constructs such as knowledge and attitude [38]. These tests verify whether the complexity of the item describes the level of cognitive ability required to obtain a correct answer. This fact has great importance for our population, since in Mexico the educational level most common among the population ≥ 15 years of age is secondary school.

Regarding discrimination, this questionnaire enables correct identification of those cases with different levels of KAP. Factor analysis results indicated the presence of at least one correlation between two items, demonstrating the feasibility of factorization. The resulting factorial matrix groups the items in three dimensions: knowledge, attitudes, and practices, just as had been proposed.

In comparison with other instruments, some of them are focused on the evaluation of KAPs during pregnancy, have been created in English [13–16], Spanish [14, 15] and Kannada [11, 12, 17] and have been used in countries such as India [11, 12, 17], Australia [18], the United States [14, 15], Latin American countries [39–41] and in some regions of Asia [10]. Despite being widely used, the domains on which the questions are conducted vary in an important way. There are studies in which only knowledge has been included, some others in conjunction with attitudes and few with the inclusion of the complete KAP elements. In addition, in the description of the analysis and the properties of the instruments, almost none of the existing instruments show validity and reliability values. Only the questionnaire used by Gupta [11], reports very similar Cronbach’s alpha and Keiser-Meyer-Olkin values to the present study. However, it should be mentioned that our instrument contains fewer questions, which is considered an advantage in terms of its application.

On the other hand, while the purpose of Gupta’s questionnaire [11] is to compare pregnant and non-pregnant women, our instrument is complemented with the use of a questionnaire on infant oral health, which provides a broader landscape in the study of maternal and infant oral health.

This work does have some limitations. First of all, it was not possible to evaluate the stability over time of the instrument. Participants were approached to evaluate the consistency of the questionnaire when they attended educational talks in the pregnant women’s club, prior to starting a maternal and child oral health program, so that once exposed to the educational sessions, it was impossible to avoid having changes in KAP. This situation essentially modified the purpose of the retest, which is to evaluate the variation from the instrument due to the effect of time when it is applied a second time under the same conditions.

Another limitation has to do with the conditions under which we had to work with the pregnant women which made it not feasible to have clinical evaluations, resulting in the impossibility to verify the validity of the criteria; it means the ability of the instrument to discriminate between the observed oral health conditions according to the variation of the instrument.

The CAPSOM questionnaire was validated in the case of Mexican women, however, with the suitable prior cultural adjustments—as pregnancy carries a significant symbolic load, we would suggest that its administration be extended to other Spanish-speaking populations. The adjustments made for each region will make it possible to improve the collection of data from each target population.

## Conclusions

The study resulted in a 10-item, self-answer questionnaire in Spanish that is valid and reliable to assess pregnant women’s oral health knowledge, attitudes, and practices, thereby contributing a valuable instrument for mother-infant wellbeing, which continues to be a priority in the area of health.

## Data Availability

The datasets used and/or analyzed during the current study are available from the corresponding author on reasonable request.

## Abbreviations

α: Cronbach’s alpha
A: Item accepted according to the value obtained from the CVR’
AAPD: American Academy of Pediatric Dentistry
CAPSOM: Maternal Oral Health Knowledge, Attitudes and Practices Questionnaire; (CAPSOM in Spanish)
CVI’: Content Validity Index
CVR’: Content validity ratio
d: Item discrimination level
D: Item difficulty index
ENES: National School of Higher Studies, Leon Unit (ENES in Spanish)
KAP: Knowledge, attitudes and practices
KMO: Kaiser–Meyer–Olkin test
R: Item rejected according to the value obtained from the CVR’
Total D: Average difficulty index of the item
UNAM: National Autonomous University of Mexico (UNAM in Spanish
